# A Case of Esophageal Cancer With Markedly Elevated Soluble Interleukin-2 Receptor: A Potential of Soluble Interleukin-2 Receptor as a Biomarker

**DOI:** 10.7759/cureus.57477

**Published:** 2024-04-02

**Authors:** Nayuta Seto, Kouichi Miura, Ling Jin, Moriyasu Nakahara

**Affiliations:** 1 Department of Internal Medicine, Chichibu Municipal Hospital, Saitama, JPN; 2 Department of Medicine, Division of Gastroenterology, Jichi Medical University, Tochigi, JPN; 3 Department of Pathology, Saitama Medical University, Saitama, JPN; 4 Department of Gastroenterology, Chichibu Municipal Hospital, Saitama, JPN

**Keywords:** tumor marker, lymph node metastasis, metastatic liver tumor, esophageal cancer, soluble interleukin-2 receptor

## Abstract

We report an autopsy case of advanced esophageal cancer with multiple metastases that presented with a markedly high level of sIL-2R. An 83-year-old man was admitted to our hospital with a 1-week history of epigastric distress, appetite loss, and fatigue. Imaging examinations revealed a large liver tumor. Although the tumor markers for gastrointestinal and liver cancers were within normal limits, the sIL-2R level was extremely high (10,384 U/mL). The patient died immediately after admission due to the rapid course of the disease. An autopsy showed advanced esophageal cancer with multiple metastases, including the liver, lungs, and multiple lymph nodes. In histological examinations, esophageal cancer was a mixture of well- and poorly differentiated squamous cell carcinoma, in which poorly differentiated cancer cells expressed sIL-2R on immunohistochemical staining. However, we failed to detect positive staining for sIL-2R in the lymphocytes. Our findings revealed that solid tumors could express sIL-2R. Although sIL-2R is a tumor marker used for hematological malignancies, such as malignant lymphoma, this case report highlights the value of the measurement of sIL-2R levels in advanced solid tumors, including esophageal cancer. We concluded that sIL-2R has potential as a biomarker in advanced solid tumors for cancer staging and treatment response.

## Introduction

Esophageal cancer is one of the common cancers in the digestive tract. Due to its aggressive nature of cancer, it is the ninth cause of cancer death in Japan [1]. Risk factors for esophageal cancer are aging, alcohol consumption, and smoking. Its common presentation includes dysphagia and weight loss. Early detection and treatment are crucial because esophageal cancer carries a risk for extensive lymph node metastasis even in an early stage. There are two histological types of esophageal cancer: squamous cell carcinoma (SCC) and adenocarcinoma. Among them, SCC is a major type of esophageal cancer in Japan. A certain number of patients with esophageal cancer are unresectable at the diagnosis. Chemoradiation therapy is a choice for patients with advanced esophageal cancer. Recently, immune checkpoint inhibitors (ICIs) were available for patients with unresectable esophageal cancer [2]. However, the prognosis of advanced esophageal cancer is still poor.

There are several types of biomarkers of esophageal cancer for detecting cancer as well as treatment response [3,4]. For instance, SCC-related antigen is used as a tumor marker for squamous cell esophageal cancer. In addition, autoantibodies, micro RNAs, metabolites, and cytokines have the potential to detect esophageal cancer [3,4].

Soluble interleukin 2 receptor (sIL-2R) is a product reflecting an immune response [5]. As a result of T cell activation, the IL-2R alpha chain (CD25), which is a portion of the IL-2 receptor, is cleaved and subsequently released into the bloodstream, resulting in sIL-2R. In cases of hemophagocytic lymphohistiocytosis or granulomatous diseases, sIL-2R levels are elevated due to an overactive immune system. The sIL-2R may also originate from the tumor itself, including malignant lymphoma, and is used as a marker for tumor load and prognosis [5]. Interestingly, serum sIL-2R is elevated in patients with metastatic gastroenterological cancers [6,7]. These data suggest that sIL-2R is a useful marker for an advanced stage of solid cancers, including esophageal cancer. In addition, sIL-2R is a potential biomarker for predicting treatment response of ICIs, which are used for second-line chemotherapy for advanced esophageal cancer [8].

We recently encountered an autopsy case of esophageal cancer with multiple metastases, in which a markedly high level of sIL-2R was noted. Thus, we should keep in mind an elevated sIL-2R level is not only a feature of hematological malignancies but also that of advanced gastrointestinal cancers. In addition, some esophageal cancer cells were positive for sIL-2R. Although it is well known that sIL-2R is produced from lymphocytes, we have little information on the sIL-2R expression in gastrointestinal cancers, including esophageal cancer. We herein reviewed the literature on the potential use of sIL-2R in gastrointestinal cancers.

## Case presentation

An 83-year-old man was referred to the emergency department with a 1-week history of mid-upper abdominal discomfort, appetite loss, and fatigue. He did not report any dysphagia or weight loss. He had a history of cerebral infarction, atrial fibrillation, chronic heart failure, hypertension, and type 2 diabetes mellitus. His medication was edoxaban, lansoprazole, furosemide, spironolactone, atenolol, alacepril, and sitagliptin. He smoked 30 cigarettes a day for 40 years, and he drank a can of beer (ethanol 14g) five times a week. He had never had an esophagogastroduodenoscopy prior to admission.

On a physical examination, he was alert and oriented. His blood pressure was 130/83 mmHg, pulse rate was 84/min, body temperature was 36.6°C, and respiratory rate was 18/min with an O_2_ saturation of 98% on ambient air. The neck, chest, abdomen, and skin were unremarkable. Neurological examination was also normal. Laboratory data on admission is shown in Table 1. Blood tests showed extremely elevated lactate dehydrogenase (LDH) of 4,741 U/L (reference range: 124 - 222) and alkaline phosphatase (ALP) of 1,276 U/L (reference range: 38 - 113) with a moderate elevation of transaminases. In addition, renal failure was noted with serum creatinine of 3.1 mg/dL (reference range: 0.65 - 1.07) and blood urea nitrogen of 92.8 mg/dL (reference range: 8 - 20). Inflammatory markers were also elevated, which was C-reactive protein of 4.7 mg/dL (reference range: 0 - 0.14). Tumor markers in the digestive system, including carcinoembryonic antigen (CEA), carbohydrate antigen 19-9 (CA19-9), α-fetoprotein (AFP), and protein induced by vitamin K absence or antagonist-II (PIVKA-Ⅱ) were normal. We, therefore, checked sIL-2R as the other tumor marker, and it was markedly elevated, 10,384 U/mL (reference range: 157 - 474).

**Table 1:** Summary of the patient's laboratory test results on admission. γ-GT: γ-glutamyl transferase; Ab: antibody; AFP:α-fetoprotein; Ag: antigen; ALP: alkaline phosphatase; ALT: alanine aminotransferase; AMA: antimitochondrial antibody; ANA: antinuclear antibody; APTT: activated partial thromboplastin time; AST: aspartate aminotransferase; CA19-9: carbohydrate antigen 19–9; CEA: carcinoembryonic antigen; CK: creatine kinase; CMV: cytomegalovirus; Cre: creatinine; CRP: C-reactive protein; EA: early antigen; EB: Epstein-Barr virus; EBNA: Epstein-Barr virus nuclear antigen; FT4: free thyroxine; HAV: hepatitis A virus; Hb: hemoglobin; HbA1c: hemoglobin A1c; HBc: hepatitis B core; HBs: hepatitis B surface; HCV: hepatitis C virus; HEV: hepatitis E virus; IgA: immunoglobulin A; IgG: immunoglobulin G; IgM: immunoglobulin M; K: potassium; LDH: lactate dehydrogenase; Na: sodium; PIVKAⅡ: protein induced by vitamin K absence or antagonist-II; PT: prothrombin time; RBC: red blood cell count; sIL-2R: soluble interleukin 2 receptor; T-Bil: total bilirubin; TP: total protein; TSH: thyroid stimulating hormone; UN: urea nitrogen; VCA: viral capsid antigen; WBC: white blood cell count

Test	Observed values	Reference range
Peripheral blood		
WBC	11,200/μL	3,900 – 9,800
RBC	403×10^4^/μL	427 – 570
Hb	13.6 g/dL	12.0 – 17.6
Platelet	19,100/μL	13,000 – 36,900
Blood coagulation factors		
PT	44.4%	70 – 140
APTT	34.7 seconds	28.5 – 40.9
D-dimer	8.9 μg/mL	0 – 1.0
Blood chemistry		
TP	6.0 g/dL	6.6 – 8.1
Albumin	2.9 g/dL	4.1 – 5.1
T-Bil	1.1 mg/dL	0.4 – 1.5
AST	241 U/L	13 – 30
ALT	127 U/L	10 – 42
LDH	4,741 U/L	124 – 222
ALP	1,276 U/L	38 – 113
γ-GT	484 U/L	13 – 64
CK	350 U/L	59 – 248
UN	92.8 mg/dL	8 – 20
Cre	3.1 mg/dL	0.65 – 1.07
Na	138 mEq/L	138 – 145
K	5.4 mEq/L	3.6 – 4.8
Cl	103 mEq/L	100 – 110
Glucose	153 mg/dL	70 – 120
HbA1c	6.5 %	4.6 – 6.2
CRP	4.7 mg/dL	0 – 0.14
Endocrine examinations		
TSH	2.48 μIU/mL	0.61 – 4.23
FT4	1.47 ng/dL	0.90 – 1.70
Tumor markers		
CEA	3.3 ng/mL	0 – 5.0
CA19-9	7.9 U/mL	0 – 37.0
AFP	2.9 ng/mL	0 – 10.0
PIVKAⅡ	24 mAU/mL	0 – 40.0
sIL-2R	10,384 U/mL	157 – 474
Serological tests		
IgG	1105 mg/dL	870 – 1700
IgA	449 mg/dL	110 – 410
IgM	73 mg/dL	33 – 190
ANA	(−)	(−)
AMA	(−)	(−)
Virological markers		
HBs-Ag	(−)	(−)
HBs-Ab	(+)	(−)
HBc-Ab	(+)	(−)
HCV-Ab	(−)	(−)
HAV-IgM	(−)	(−)
HAV-IgG	(+)	(−)
HEV-IgA	(−)	(−)
CMV-IgM	(−)	(−)
CMV-IgG	(+)	(−)
EB VCA-IgM	(−)	(−)
EB VCA-IgG	(+)	(−)
EB EA-IgG	(−)	(−)
EBNA-Ab	(+)	(−)
Urine tests		
Protein	(−)	(−)
Occult blood	(−)	(−)
WBC	(−)	(−)

**Figure 1:**
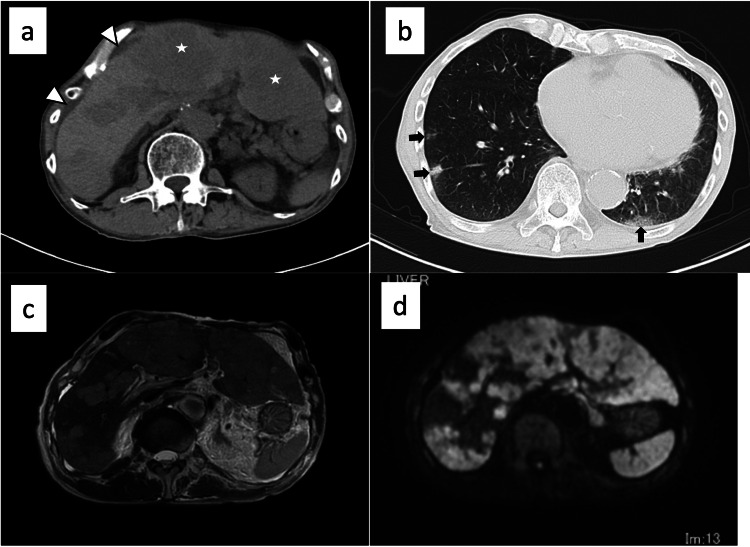
Imaging findings. Non-contrast computed tomography (CT) image (a, b). (a) Abdominal CT scan revealed low-density lesions in the liver (stars), mainly left but also right lobe, and a small amount of ascites (arrowheads). These findings suggested malignant tumors, including metastatic liver tumors. (b) Chest CT scan showed multiple ground-glass opacity and infiltrate shadows in the bilateral peripheral lungs (black arrows), suggesting metastatic lung tumors. Non-contrast magnetic resonance imaging (c, d). Most of the liver was replaced by neoplastic lesions. There was no evidence of intrahepatic bile duct dilation. (c) T2-weighted image; (d) diffusion-weighted image.

A non-contrast computed tomography (CT) scan showed low-density lesions in the liver, a small amount of ascites, and multiple ground-glass opacity and infiltrate shadows in the bilateral peripheral lungs (Figures 1a-1b). No splenomegaly and lymphadenopathy were observed. Abnormal findings of the esophagus were not pointed out. In a non-contrast magnetic resonance imaging (MRI), the liver tumor was characterized by T1-hypointense, T2-hyperintense, and diffusion-weighted imaging-hyperintense. There was no evidence of intrahepatic bile duct dilation (Figures 1c-1d). Although an elevation of sIL-2R level prompted us to assess the disease, including malignant lymphoma, respiratory failure and consciousness disturbance occurred on the fifth day of hospitalization. We failed to assess the gastrointestinal tract and the liver using esophagogastroduodenoscopy and liver biopsy due to the urgent course of the disease. He unfortunately died on the sixth day of hospitalization.

**Figure 2:**
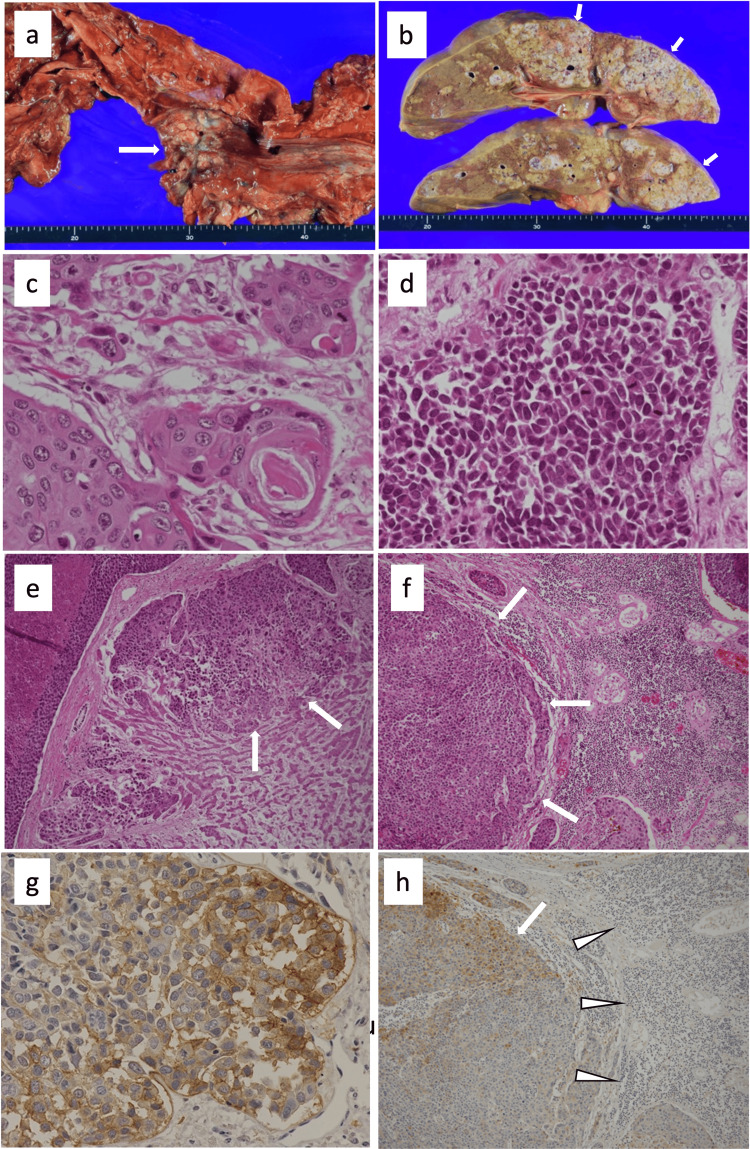
Macrograph findings (a,b) and histopathological findings (c-h). (a) There is a 60 × 50 mm tumor (arrow) in the middle intrathoracic esophagus. The tumor appears ulcerated, (b) over 80% of the liver was occupied with tumor (arrows). The esophageal tumor was a mixture of (c) well-differentiated and (d) poorly differentiated squamous cell carcinoma (Hematoxylin & Eosin staining, ×40). (e) Squamous cell carcinoma was observed in the liver with portal vein invasion (arrows) (Hematoxylin & Eosin staining, ×10). (f) Squamous cell carcinoma was observed in the lymph node (arrows) (Hematoxylin & Eosin staining, ×10). (g) Immunohistochemical staining for CD25 in the esophageal cancer. CD25 is the interleukin 2 receptor (IL-2R) alpha chain, which is a component part of IL-2R. Because the soluble interleukin 2 receptor (sIL-2R) is secreted by CD25, its positivity implies sIL-2R expression. Poorly differentiated cancer cells are positive for CD25 (×40). (h) Immunohistochemical staining for CD25 in the lymph node. CD25 staining was positive only in tumor cells (arrow) but negative in lymphocytes (arrowheads) (×40).

In an autopsy, there was a tumor, suspicious of the advanced cancer, in the mid-portion of the esophagus (Figure 2a). In addition, over 80% of the liver was filled with tumor (Figure 2b). The lungs exhibited bilateral diffuse hemorrhagic and edema. Histopathological examinations demonstrated that the esophageal tumor was a mixture of well- and poorly differentiated SCC (Figures 2c-2d). The histological assessment of the liver tumor was also SCC (Figure 2e). In addition, SCC was observed in the alveolar space of the lungs. Tumor embolisms were noted in the portal vein and in the pulmonary artery, which caused necrosis of the liver and bleeding of the lungs, respectively. Tumor embolism in the pulmonary artery was suspected as a cause of death. There were multiple lymph node metastases (Figure 2f). There was no evidence of malignant lymphoma or inflammatory diseases, including sarcoidosis. Based on histological examination and the distribution of tumor cells, we concluded that the primary lesion was the esophagus and that metastatic lesions were involved in the liver, the lung, and the lymph nodes.

Immunohistochemical studies showed that a certain number of cancer cells in the esophagus and metastatic lesions, including the liver and the lymph nodes, were positive for CD25, a synonym of sIL-2R (Figure 2g). The proportion of CD25-positive cells was less than 5% of all cancer cells, and most of the positive cells were observed in poorly differentiated carcinoma but not well-differentiated carcinoma. However, we failed to detect positive cells in normal portions of the lymph nodes (Figure 2h).

## Discussion

We herein reported a case of advanced esophageal cancer with a markedly high level of sIL-2R. Serum sIL-2R is well-known as a marker for malignant lymphoma. In the present case, there were no findings of hematological malignancies in the bone marrow and the lymph nodes. In addition, there was no evidence of inflammatory diseases, including sarcoidosis, which can elevate the sIL-2R level. Furthermore, the tumors in the liver and the lungs were metastatic lesions from esophageal cancer. Thus, we concluded that the primary cause of marked elevation of sIL-2R level was esophageal cancer.

Esophageal cancer, particular in cases with metastatic lesions, has been reported to show a high level of sIL-2R in the serum. Murakami reported that sIL-2R levels in patients with esophageal cancer were higher than those in health subjects (490 vs 276 U/mL) [6]. Wang et al. revealed that sIL-2R levels in patients with esophageal cancer was 1.5-1.7 times higher than those in healthy subjects [7]. Other cancers, including gastric cancer and colorectal cancer, are also reported to show elevated sIL-2R levels, with 442-491 U/mL in average [6]. These findings indicate that sIL-2R elevates in patients with solid cancers. Serum sIL-2R further elevates in patients with metastatic lesions. Thus, sIL-2R is proposed as a biomarker for an advanced stage of gastrointestinal cancers. Although renal impairment can cause an elevated sIL-2R level [9], it is difficult to account for extremely high levels of sIL-2R (10,384 U/mL) by renal dysfunction.

We then attempted to examine the potential source of sIL-2R using immunohistochemistry. As a result, we showed that a part of esophageal cancer cells expressed CD25, suggesting that they produced sIL-2R. To our knowledge, this is the second report that esophageal cancer cells express sIL-2R at the protein level [7]. The mechanism by which esophageal cancer expresses sIL-2R remains unknown. A former report showed that there was a trend between poorly differentiated esophageal cancer and sIL-2R levels [7]. In our case, poorly differentiated esophageal cancer expressed CD25, suggesting that there is a potential association between cancer differentiation and sIL-2R expression. Other than non-gastrointestinal cancers, breast cancer cells express sIL-2R [6]. In general, sIL-2R is produced by lymphocytes, including T cells and B cells. In lymph node metastasis, lymphocytes are activated by tumor cells, leading to the production of sIL-2R. In a microenvironment of tumors, elevated IL-2, a ligand for IL-2R, combines with its specific receptor, then the α-chain of IL-2R is released into the peripheral bloodstream as a soluble form [6]. Thus, it is convincing that metastatic tumors can elevate serum sIL-2R. In contrast to the mechanism previously reported, the lymphocytes in the lymph node were negative for sIL-2R in our case, suggesting that sIL-2R production by esophageal cancer cells may have resulted in the high sIL-2R levels, even in the absence of metastasis or advanced stage. However, we cannot deny the possibility that lymphocytes produce sIL-2R from non-examined organs.

The significance of sIL-2R in solid tumors remains unknown. Recently, sIL-2R has been reported as a potential biomarker for immune-related adverse events as well as response to ICIs. In patients who had immune-related adverse events, serum sIL-2R was elevated [10]. In non-small cell lung cancer, patients with a high level of sIL-2R failed to continue immunotherapy; no patients continued longer than 9.3 months, which is in contrast to patients with a low level of sIL-2R [8]. As a result, progression-free survival was shorter in patients with a high level of sIL-2R. In addition, patients with a high level of IL-2 showed thyroid dysfunction during the treatment of ICIs [11]. Because a high level of sIL-2R reflects high levels of IL-2, which is difficult to measure due to its short life span. These findings suggest that sIL-2R may serve as a biomarker for an adverse response to ICIs and that measurement of sIL-2R levels should be considered when treating with ICIs. Further investigations are necessary to prove this hypothesis.

Although the present case showed a markedly high level of sIL-2R, the blood test was carried out only once in a terminal stage. Thus, an unexpected immune response at the terminal stage may elevate the sIL-2R in our case. In addition, we did not have a chance to cancer treatment due to the rapid course of the disease. Thus, further investigations are necessary to prove the association between treatment response and sIL-2R. However, our case sheds light on the measurement of sIL-2R levels in advanced esophageal cancer.

## Conclusions

We experienced the case of advanced esophageal cancer, in which cancer cells express sIL-2R with an extremely high level of sIL-2R. The present patient was a distinctive case from former reports, in which high sIL-2R levels were caused by activation of lymphocytes. Further research is needed to determine the utility of serum sIL-2R levels and nature of sIL-2R expressing cancers in solid tumors.
